# Exploring Microstructure Measures and Topics in Personal Narratives of Turkish School‐Age Children: Findings From the Global TALES‐TR Protocol

**DOI:** 10.1111/1460-6984.70219

**Published:** 2026-03-08

**Authors:** Esra Yaşar Gündüz, Semra Selvi Balo, İlknur Maviş

**Affiliations:** ^1^ Department of Speech and Language Therapy Cappadocia University, School of Health Sciences Nevsehir Türkiye; ^2^ Department of Speech and Language Therapy, Faculty of Health Sciences Anadolu University Eskisehir Türkiye; ^3^ President of the Association for Turkish Speech and Language Therapists (DKTD) Eskisehir Türkiye

**Keywords:** global TALES protocol, microstructure, personal narrative, SALT

## Abstract

**Background:**

The Global TALES protocol is an internationally used personal narrative assessment tool designed to elicit and analyse children's personal narratives across languages and cultures.

**Aim:**

The aim of this study is to provide the Turkish literature with assessment data derived from typically developing (TD) children aged 7–10 years and to examine the applicability of the Turkish‐adapted version within a Turkish linguistic and cultural context.

**Methods and Procedures:**

This descriptive, cross‐sectional study included eighty monolingual Turkish‐speaking TD children, with 20 children in each age group (7–10 years). The Turkish‐adapted Global TALES‐TR protocol was conducted online with the children. The narratives were assessed for microstructure measures using the Turkish Research Version of the Systematic Analysis of Language Transcripts (SALT) program. SPSS 22.0 and TURCOSA software were used for statistical analysis.

**Results:**

Some age‐related differences were observed in children's narrative microstructure, with significant contrasts between the youngest and oldest groups in verbal productivity and semantic diversity, while a more limited age effect was observed for semantic diversity between the age‐8 and age‐9 groups. Gender‐related differences were limited. When the full sample was considered, a measure reflecting syntactic complexity showed a gender effect. Age‐specific analyses indicated that gender‐related differences in verbal productivity and syntactic complexity were observed only in the age‐9 group. All observed gender‐related differences favoured girls. The frequency of follow‐up prompts and unanswered prompts showed that the ‘problem’ narrative was the most challenging for children. Narratives primarily focused on topics such as family, friends, school and success.

**Conclusions:**

The results show that the adapted protocol can be used in personal narrative assessment. These findings may inform future research and clinical assessment practices. Furthermore, using this protocol, broader information can also be gathered about groups such as those with language disorders.

**WHAT THIS PAPER ADDS:**

*What is already known on the subject*

- Personal narratives provide rich information about children's language, discourse and communicative competence and are widely used in both research and clinical assessment. The Global TALES protocol has been developed as a cross‐linguistic and cross‐cultural framework to elicit personal narratives using emotion‐based prompts and has been applied in multiple languages and cultural contexts. However, empirical evidence on personal narrative performance in Turkish‐speaking children remains limited, and systematic data obtained using internationally comparable protocols are scarce.

*What this study adds to existing knowledge*

- This study extends a previous pilot study by providing systematically analysed assessment data from a larger sample of TD Turkish‐speaking children aged 7–10 years using the Turkish‐adapted Global TALES protocol. The findings offer detailed information on verbal productivity, semantic diversity and syntactic complexity in Turkish personal narratives, including age‐related patterns and limited gender‐related differences. In addition, the study documents children's use of follow‐up prompts and the narrative topics elicited by different emotional prompts, contributing cross‐cultural data that can be compared with findings from other countries participating in the Global TALES project.

*What are the potential or actual clinical implications of this work?*

- The results provide reference data that may support speech‐language pathologists in interpreting personal narrative samples from Turkish‐speaking children aged 7–10 years. The study demonstrates the feasibility of using the Global TALES protocol in Turkish, offering clinicians a culturally sensitive and internationally comparable framework for narrative assessment. The findings may inform clinical decision‐making, assessment planning and future research involving children with language disorders or learning difficulties.

## 1 Introduction

Narrative is a type of discourse that reflects overall language ability and indicates communicative competence (Boudreau 2008; McCabe and Marshall 2006; Botting 2002). The literature has predominantly focused on fictional narratives, which are typically elicited using pictorial stimuli (e.g., Gagarina et al. 2012; Mayer 1969). Personal narratives, another kind of narrative, are descriptions of past events experienced by the speaker (McCabe and Bliss 2003) and are considered the earliest and most essential form of narrative acquired by children (McCabe et al. 2008). Personal narratives offer detailed insights into skills such as attention, logical sequencing and the ability to connect events to a schema (Griffith et al. 1985; John 2001; Sarbin 2004). Moreover, personal narratives form the foundation of social interaction, help create memories and experiences, and play a vital role in academic success (Reed and Spicer 2003; Westby and Culatta 2016; Westerveld and Claessen 2014). Additionally, personal narratives reflect the cultural style of society, as narrative expectations and structures may vary across cultures, with some emphasising brevity and others favouring more elaborated storytelling. These differences may influence event sequencing, background information and microstructure measures (Bliss and McCabe 2008; Carmiol and Sparks 2014).

In order to better understand cross‐cultural differences and consider these factors during assessment and therapy, the use of cross‐culturally applicable assessment tools is of great importance (McLeod and Threats 2008; Westerveld et al. 2022). To provide opportunities for cross‐linguistic and cross‐cultural research, members of the Child Language Committee of the International Association of Communication Sciences and Disorders (IALP) developed the Global TALES (Talking About Lived Experiences in Stories) protocol (Westby 2021; Westerveld 2020; Westerveld et al. 2020). This project aims to investigate effective ways to elicit and analyse personal narratives from children and to provide a practical protocol for clinicians worldwide (Westerveld 2020; Westerveld et al. 2021).

Using the Global TALES protocol, personal narratives have been elicited from children and adolescents across various countries, including New Zealand, Australia, Greece, Russia, Taiwan and the United Kingdom. These studies have explored narratives from different perspectives, such as topic variation, macro‐ and microstructure analysis parameters, and cross‐cultural comparisons of narrative topics (e.g., Westby et al. 2023; Westerveld et al. 2022; Westerveld et al., 2023).

### 1.1 Study Purpose and Research Questions

Narrative‐based language samples from children aged four or five and older are highly informative, as even relatively brief oral narratives capture children's language use in a natural communicative context, encompassing both microstructure features (e.g., semantic diversity and syntactic complexity) and macrostructure aspects such as narrative organisation and event sequencing (Heilmann et al. 2010a, Heilmann et al. 2010b; Miller et al. 2015). Despite this, personal narrative assessment has received limited attention in the literature, and there is no standardised assessment tool available in Turkish. A previous study examining the personal narratives of Turkish‐speaking children focused on narratives elicited in relation to the emotion of ‘fright’. In this study, Akıncı‐Oktay (2010) analysed the linguistic and sociolinguistic structure of narratives elicited from 200 children aged 9 to 10 and compared them based on parental education level. The children were asked to write about their most frightening experience. The results showed that children in the high parental education group produced more words and sentences in their narratives than those in the low parental education group.

More recently, the Global TALES Protocol was adapted into Turkish and preliminarily examined in a pilot study conducted with typically developing (TD) children (Maviş and Yaşar‐Gündüz 2023). This pilot study supported the feasibility of administering the protocol in Turkish and informed minor refinements. Building on this preliminary adaptation, the present study extends this study by examining the applicability of the Turkish‐adapted Global TALES protocol in a larger and more systematically analysed sample of TD children. Accordingly, the primary aim of the current study is to report detailed assessment outcomes obtained using the Turkish‐adapted Global TALES in a systematically analysed sample of TD Turkish‐speaking children. The findings obtained from TD children are expected to serve as a foundation for assessing children with language disorders and/or learning difficulties (Westerveld 2020; Westerveld et al. 2021), thereby contributing to the evaluation and intervention processes of speech‐language pathologists.

The Global TALES protocol was developed as an international narrative elicitation framework designed to examine children's personal narrative abilities in a comparable manner across different languages and cultures (Westerveld et al. 2022). The primary reason for selecting Global TALES in the present study is that the protocol was specifically designed to minimise cultural and linguistic bias. This is achieved through the use of open‐ended, emotion‐based prompts that allow children to select and recount meaningful experiences from their own lives, thereby reducing culturally constrained responding. Accordingly, Global TALES functions not only as a tool for assessing narrative performance but also as a framework that supports the generation of normative data across languages and enables meaningful clinical comparisons. In this context, adapting the Global TALES protocol into Turkish and examining its applicability in Turkish‐speaking children aims to contribute to the development of a systematic and internationally comparable approach to the assessment of personal narrative abilities in Turkish‐speaking populations.

The following research questions (RQ) are addressed within the scope of this study:

RQ‐1 How do TD children perform on microstructure measures (verbal productivity, semantic diversity, syntactic complexity) in response to the Global TALES‐TR protocol?
RQ‐2 Do the microstructure performance results of TD children show statistically significant differences based on age and gender variables?
RQ‐3 What is the frequency of eliciting personal narratives through the Global TALES‐TR protocol prompts?
  a. How frequently do TD children require follow‐up prompts when responding to the prompts?
  b. What is the frequency of prompts for which personal narratives could not be elicited from TD children?
RQ‐4 Which topics are most commonly mentioned in the personal narratives of TD children?

## 2 Method

Ethical approval for participating in the project team and using the Global TALES protocol in research has been granted by the Griffith University Human Research Ethics Committee (GUHREC, 2020/942) and the Anadolu University Scientific Research and Publication Ethics Committee of the Faculty of Health Sciences (Protocol No: 358190).

The study was designed as a descriptive cross‐sectional research model comparing microstructure measures based on different variables. The independent variables in the research were age and gender, while the dependent variables were the microstructure measures elicited from the children's personal narratives.

### 2.1 Participants

The study involved 80 TD monolingual Turkish‐speaking children aged 7 to 10 years. The sample size was based on prior research (Lyons et al. 2023; Srivastava et al. 2023; Theodorou et al. 2023). Inclusion criteria specified that children (i) had no history of language or speech disorders, (ii) were not receiving special education, occupational therapy, or other support services during the study, and (iii) had no sensory, cognitive, or motor developmental problems. To prevent children from receiving cues from others or being influenced by a parent's presence, and to ensure a quiet testing environment, each child participated in the online session alone in a room in their home. Out of 86 children initially interviewed, six were excluded for not meeting these conditions. Descriptive characteristics of the participants are presented in Table 1.

**TABLE 1:** Descriptive characteristics of the participants.

		Age 7	Age 8	Age 9	Age 10
**Gender (*n*)**	Female	10	10	10	10
	Male	10	10	10	10
**Age**	Mean ± SD	7;5 ± 0;4	8;6 ± 0;4	9;6 ± 0;4	10;4 ± 0;4
	Min–max	7;0–7;11	8;0–8;11	9;0–9;11	10;0–10;11
**Parent education (*n*)**	**Mother**				
	Primary/middle school	4	6	5	5
	High school	3	4	3	7
	Bachelor	13	7	11	5
	Postgraduate	—	3	1	3
	**Father**				
	Primary/middle school	3	4	2	2
	High school	4	5	4	7
	Bachelor	12	8	11	9
	Postgraduate	1	3	3	2
**Income level (*n*)**	Very low	1	—	—	1
	Low	1	1	—	1
	Average	15	16	18	14
	High	3	3	2	4

### 2.2 Data Collection Tools

#### 2.2.1 Global TALES Protocol

The Global TALES protocol consists of two parts: a demographic questionnaire and a protocol form. Through the Global TALES demographic form, information such as the child's age, gender and academic performance is gathered. In this study, the form was filled out using information provided by the parent, usually the mother.

The protocol includes types of emotions and events that are relevant and applicable to children worldwide (Westerveld 2020; Westerveld et al. 2021; Westerveld et al. 2022). It consists of six prompts in the following order: ‘Happy/excited’, ‘Worried/confused’, ‘Annoyed/angry’, ‘Proud’, ‘Problem’ and ‘Important’. Each is accompanied by one follow‐up prompt. For example, if a child cannot respond within 10 seconds to the prompt, ‘Tell me a story about something that has happened to you that was very important to you,’ the follow‐up prompt—‘Some children tell me about winning something, or maybe a time when they did very well at school’—is used.

### 2.3 Procedure and Measures

#### 2.3.1 Adaptation Process to Turkish

During the adaptation of the protocol into Turkish, a meaning‐based translation approach was adopted with careful attention to linguistic and cultural appropriateness. The protocol was translated by bilingual experts and finalised through researcher consensus. The Turkish version was subsequently reviewed by field experts to ensure clarity and suitability for Turkish‐speaking children, and the final version was established accordingly (see Maviş and Yaşar Gündüz 2023). As the Global TALES protocol was originally developed to be applicable across diverse cultural contexts, no substantial difficulties were encountered during the adaptation process.

#### 2.3.2 Pilot Study Process

A pilot study was conducted from 15 November to 31 December 2022, using the adapted version of the protocol to assess comprehensibility and identify potential issues that might arise during implementation (Hazzi and Maldaon 2015). This pilot involved a separate group of children from those included in the main study. Following the pilot study, revisions were made to the demographic questionnaire form (Maviş and Yaşar‐Gündüz 2023), and the final version of the Turkish adaptation of the protocol was completed in February 2023.

#### 2.3.3 Data Collection Process

Participants were reached through personal and social networks using snowball and purposive sampling methods. The interviews were conducted online (via Zoom) in a single session lasting about 30 min, which included the collection of background information from parents and the narrative elicitation tasks. Online administration was selected to facilitate access to participants from all seven geographical regions of Türkiye and to enhance the representativeness of the sample. All sessions were conducted by the first author of the study (E.Y.G.), who is a speech‐language pathologist.

First, demographic forms were completed through interviews with the parents. The children's personal narratives were also elicited during the same session, while the children were at home and alone in their rooms. To ensure rapport and child engagement in the online setting, several strategies were employed. To help the child feel comfortable with the researcher (Miller et al. 2020), a warm‐up conversation lasting at least five minutes was conducted with each child; then, the protocol prompts were presented on the computer screen and directed to the child. As noted in the literature (Westerveld et al. 2020, Westerveld et al. 2021), the researcher showed interest in the child's storytelling through facial expressions and non‐directive encouragements (such as ‘oww!’ or ‘mm‐hmm’). When the narrative was brief or no response was received, follow‐up prompts or additional encouragements from the protocol were used to encourage further storytelling.

### 2.4 Data Analysis

#### 2.4.1 Language Sample Analysis

Microstructure analysis serves as an indicator that reveals the strengths and weaknesses of different language components (Hughes et al. 1997; Westerveld and Gillon 2010). In this study, the focus was on microstructure measures of personal narratives, and as suggested (Westerveld et al. 2020), the Systematic Analysis of Language Transcripts (SALT) software was used (Acarlar et al. 2020). Only completed and intelligible verbal utterances were included in the analysis, and certain measures were calculated accordingly: Verbal productivity (*Total Number of Utterances: NTU; Total Number of Words: TNW*)—Semantic diversity (*Number of Different Words: NDW; Moving Average Type‐Token Ratio: MATTR*)—Syntactic complexity (*Mean Length of Utterance in morphemes (MLU‐M) and in words (MLU‐W*)).

NTU and TNW measures indicate verbal productivity (Leadholm and Miller 1994; Miller 1991). At this stage, key transcription conventions were carefully followed in the transcription of utterances and words (Acarlar and Johnston 2006; Miller et al. 2020). For example, filler words (such as ‘ee’ and ‘ıı’) and revisions were placed in parentheses and excluded from the analysis.

NDW, an indicator of semantic diversity (Leadholm and Miller 1994; Miller et al. 2020), is based on calculating different word roots (Acarlar 2005). At this stage, relevant transcription rules were also followed (Acarlar and Johnston 2006; Miller et al. 2020). For example, attention was paid to root changes caused by affixes, and words were transcribed in their base or root forms (e.g., ‘gidiyorum’ = git/iyor/um).

MATTR measures semantic diversity by averaging consecutively calculated type‐token ratios (Acarlar 2024). In this study, MATTR was calculated using a moving window of 100 consecutive words.

MLU, which is considered an important developmental measure (Leadholm and Miller 1994), provides information based on syntactic complexity. In this study, as another measure, MLU was calculated for both morphemes and words in the narratives.

The recordings were reviewed and transcribed by the study's first author (E.Y.G.). For transcription reliability (Shriberg et al. 1984), 30% of the narratives were independently transcribed again by an expert with a PhD in speech and language therapy who was not involved in the initial transcription. Reliability was confirmed through consensus (utterances: 90.28%; morphemes: 94.35%).

Finally, narrative topics and example contents were identified through consensus between the study's first author and an expert. For each prompt, the three most frequently mentioned topics were identified. The main topics in the present study were addressed by integrating relevant studies (Lyons et al. 2023; Srivastava et al. 2023; Westby et al. 2023; Westerveld et al. 2022).

#### 2.4.2 Statistical Analysis

Statistical analyses were performed using SPSS Statistics 22.0 and TURCOSA software. Descriptive statistics for participants’ demographic information and microstructure measures were presented as means, standard deviations (SD), medians (med), minimums (min) and maximums (max). Measure results were mainly compared based on age and gender variables.

The normality of data distributions was evaluated using the Shapiro–Wilk test; parametric tests and non‐parametric tests were applied based on distribution characteristics. Analyses were conducted as within‐group or between‐group comparisons; the significance level was set at *p* < 0.05 for all analyses. When overall analyses involving more than two groups yielded significant results, post hoc analyses were conducted to determine the specific group differences underlying the observed effects. Assumptions such as normality and homogeneity of variance were considered during post hoc comparisons. Effect sizes (Cohen 1988; Tomczak and Tomczak 2014) were calculated to quantify the magnitude of age‐ and gender‐related differences.

## 3 Results

This study aimed to (i) describe the microstructural characteristics of personal narratives elicited using the Global TALES‐TR protocol, (ii) examine age‐ and gender‐related differences in microstructural measures, (iii) assess follow‐up prompt use and instances in which narratives could not be elicited, and (iv) identify the most frequently mentioned narrative topics. The results from the data analysis are presented in accordance with these research objectives.

### 3.1 Descriptive Analysis Results of Microstructure Measures

Within the scope of the study's first research question, personal microstructure measures elicited from TD children were analysed descriptively. Descriptive statistics for each age group's microstructure measures are presented in Table 2.

**TABLE 2:** Descriptive analysis results of microstructure measures across different age groups.

	Age groups	*n* ^*^	Mean	SD	Med	Min	Max
**Total Number of Utterance (NTU)**	Age 7	20	47.40	30.05	38.5	12	110
	Age 8	20	63.70	54.08	45.5	16	234
	Age 9	20	74.20	56.23	53.5	17	271
	Age 10	20	89.15	62.84	70.5	30	277
**Total Number of Words (TNW)**	Age 7	20	213.90	124.62	184	55	494
	Age 8	20	294.75	230.91	199	89	1000
	Age 9	20	359.25	292.02	239.5	95	1366
	Age 10	20	445.70	308.66	352	107	1319
**Number of Different Words (NDW)**	Age 7	20	98.20	40.82	91.5	44	179
	Age 8	20	118.45	61.12	99	53	281
	Age 9	20	144.00	64.62	123	43	325
	Age 10	20	166.60	78.53	155	67	340
**Moving Average Type‐token Ratio (MATTR)**	Age 7	17	0.59	0.05	0.61	0.44	0.67
	Age 8	19	0.58	0.04	0.59	0.51	0.66
	Age 9	19	0.63	0.03	0.63	0.57	0.69
	Age 10	20	0.61	0.04	0.62	0.52	0.70
**MLU‐in words (MLU‐W)**	Age 7	20	4.67	0.76	4.62	3.50	6.53
	Age 8	20	4.77	0.45	4.68	4.04	5.79
	Age 9	20	4.78	0.61	4.54	3.81	5.94
	Age 10	20	4.97	0.75	4.8	3.45	6.32
**MLU–in morphemes (MLU‐M)**	Age 7	20	9.20	1.69	9.02	6.85	14.00
	Age 8	20	9.24	1.04	9.43	6.98	10.92
	Age 9	20	9.21	1.02	9.06	7.47	11.47
	Age 10	20	9.53	1.34	9.05	7.55	12.22

* Data from 20 children in each age group were included. However, in the MATTR measure, data loss occurred due to the inability to elicit a narrative with at least 100 words from some participants.

As shown in Table 2, the mean and median values of NTU, TNW and NDW measures gradually increased with age, with the highest values observed in the age‐10 group. In contrast, the mean and median values of MLU‐W, MLU‐M and MATTR were closely aligned across all age groups.

### 3.2 Comparative Analysis of Microstructure Measures by Age and Gender

In accordance with the second research question, a statistical analysis of microstructure measures elicited from TD children's narratives was performed based on age and gender variables.

#### 3.2.1 Comparative Analysis by Age Groups

**FIGURE 1:**
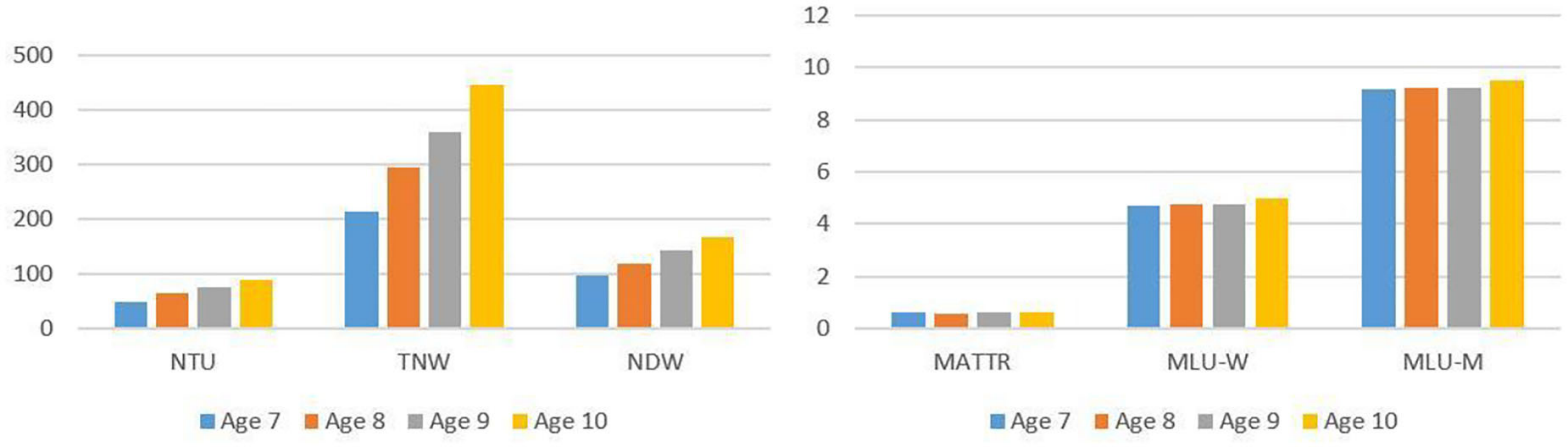
Distribution of microstructure measures across age groups.

The study includes four age groups, each consisting of 20 children. Figure 1 shows the distribution of microstructure measures derived from the children's narratives across these age groups.

Based on Figure 1, descriptively higher values were observed in the age‐10 group across all microstructure measures except MATTR, whereas the lowest NTU, TNW and NDW scores were observed in the age‐7 group.

According to the Shapiro–Wilk test, NTU, TNW and NDW measures did not follow a normal distribution (*p* < 0.05), while MLU‐W, MLU‐M and MATTR measures were normally distributed (*p* > 0.05). Accordingly, one‐way ANOVA was used to analyse differences between groups for normally distributed data, whereas the Kruskal–Wallis test was applied for non‐normally distributed data (see Table 3).

**TABLE 3:** Findings from the comparison of microstructure measures across age groups.

Measures	(*X* ^2^) / *F*	Effect size (*ϵ* ^2^ / *η* ^2^)	*p*	Post hoc analysis	
				Groups	*p*
**NTU**	9.088	**0.12**	0.028*	1–4	0.028*
**TNW**	9.398	**0.12**	0.024*	1–4	0.020*
**NDW**	12.527	**0.16**	0.006*	1–4	0.007*
**MLU‐W**	0.695	0.03	0.558	—	—
**MLU‐M**	0.302	0.01	0.824	—	—
**MATTR**	3.200	**0.12**	0.028*	2–3	0.027*

*p* < .05; Kruskal–Wallis test statistics (*χ* ^2^) and effect size (*ϵ* ^2^) are reported for non‐parametric comparisons; *F* values and effect size (*η* ^2^) are reported for ANOVA; Groups: Age 7 (1)—Age 8 (2)—Age 9 (3)—Age 10 (4).

The Kruskal–Wallis test revealed significant differences among age groups in NTU (*p* = 0.028, *ϵ* ^2^ = 0.12), TNW (*p* = 0.024, *ϵ* ^2^ = 0.12) and NDW (*p* = 0.006, *ϵ* ^2^ = 0.16) measures. The Bonferroni‐corrected post hoc analysis indicated that these differences were solely due to the contrast between the age‐7 and age‐10 groups (see Table 3). Accordingly, the mean ranks of the age‐7 group for these measures were significantly lower than those of the age‐10 group, with effect sizes in the moderate range for NTU and TNW and a large effect for NDW.

One‐way ANOVA indicated a significant difference among age groups only for the MATTR (*p* = 0.028, *η* ^2^ = 0.12). According to the results of the Tukey test, which was conducted assuming homogeneity of variance (*p* = 0.461), this difference was observed only between the age‐8 and age‐9 groups (*p* = 0.027); the mean MATTR score of the age‐8 group was significantly lower than that of the age‐9 group, representing a moderate effect size.

#### 3.2.2 Comparative Analysis by Gender Groups

Gender‐based comparisons were conducted for the entire sample and across age groups, with descriptive results shown in Table 4.

**TABLE 4:** Descriptive analysis results of microstructure measures for female (F) and male (M) children.

		*N*		Mean		SD		Med		Min		Max	
		F	M	F	M	F	M	F	M	F	M	F	M
**Age 7**–**10**	**NTU**	40	40	74.85	62.37	65.27	38.26	49.00	53.00	12	16	277	177
	**TNW**	40	40	363.02	293.77	313.29	189.04	243.00	239.50	55	89	1366	848
	**NDW**	40	40	137.80	125.82	75.41	57.25	120.00	116.00	44	43	340	301
	**MATTR**	38	37	0.60	0.61	0.04	0.05	0.60	0.62	0.53	0.44	0.70	0.69
	**MLU‐W**	40	40	4.91	4.68	0.58	0.71	4.85	4.59	3.77	3.45	6.13	6.53
	**MLU‐M**	40	40	9.45	9.13	1.08	1.46	9.47	8.90	7.53	6.85	11.91	14.00
**Age 7**	**NTU**	10	10	42.9	51.9	28.64	32.26	34.5	45	12	19	91	110
	**TNW**	10	10	191.4	236.4	104.40	144.08	182.5	207	55	91	360	494
	**NDW**	10	10	89.8	106.6	35.34	45.94	89.5	98.5	44	60	138	179
	**MATTR**	8	9	0.59	0.60	0.04	0.07	0.59	0.62	0.53	0.44	0.67	0.67
	**MLU‐W**	10	10	4.74	4.60	0.70	0.85	4.62	4.58	3.78	3.5	5.82	6.53
	**MLU‐M**	10	10	9.53	8.86	1.32	2.01	10.10	8.65	7.53	6.85	10.93	14
**Age 8**	**NTU**	10	10	69.4	58	63.77	45.11	39.5	51	20	16	234	164
	**TNW**	10	10	324.2	265.3	267.81	197.26	189.5	224	102	89	1000	712
	**NDW**	10	10	126.6	110.3	68.75	54.87	96.5	100	60	53	281	225
	**MATTR**	10	9	0.59	0.59	0.04	0.04	0.59	0.59	0.53	0.51	0.66	0.64
	**MLU‐W**	10	10	4.88	4.66	0.45	0.44	4.79	4.6	4.27	4.04	5.79	5.56
	**MLU‐M**	10	10	9.41	9.05	0.84	1.23	9.48	8.86	7.94	6.98	10.52	10.92
**Age 9**	**NTU**	10	10	93.9	54.5	72.03	24.99	75	50	35	17	271	111
	**TNW**	10	10	476.3	242.2	370.09	112.33	360.5	215	159	95	1366	473
	**NDW**	10	10	171.6	116.4	76.36	35.94	154.5	114.5	94	43	325	176
	**MATTR**	10	9	0.62	0.64	0.03	0.04	0.62	0.66	0.57	0.59	0.66	0.69
	**MLU‐W**	10	10	5.05	4.50	0.56	0.55	5.1	4.40	4.34	3.81	5.94	5.59
	**MLU‐M**	10	10	9.30	9.10	0.95	1.13	9.31	8.97	8.09	7.47	10.73	11.47
**Age 10**	**NTU**	10	10	93.2	85.1	80.27	43.09	59	79.5	30	31	277	177
	**TNW**	10	10	460.2	431.2	382.87	232.52	307.5	378.5	113	107	1319	848
	**NDW**	10	10	163.2	170	90.27	69.60	136.5	171.5	78	67	340	301
	**MATTR**	10	10	0.60	0.61	0.04	0.04	0.60	0.63	0.55	0.52	0.7	0.67
	**MLU‐W**	10	10	4.99	4.94	0.61	0.91	4.86	4.74	3.77	3.45	6.13	6.32
	**MLU‐M**	10	10	9.56	9.49	1.28	1.46	9.19	8.84	7.63	7.55	11.91	12.22

Normality assumptions were evaluated using the Shapiro–Wilk test. For data with a normal distribution (*p* > 0.05), independent samples *t*‐tests were performed, and the results were reported with the mean difference, *t*, *df*, effect size (*d*) and *p* values. For data that were not normally distributed (*p* < 0.05), the Mann–Whitney *U* test was used, and the results were presented with mean ranks (female‐male), *U*, *z*, effect size (*r*) and *p* values (see Table 5).

**TABLE 5:** Comparison findings of microstructure measures between female (F) and male (M) children.

Groups	Measure	Mean difference / Mean ranks (F − M)	*t* / *U*	*df* / *z*	Effect size (*d* / *r*)	*p*
**Age 7**–**10**	**NTU**	40.81 − 40.19	787.50	−0.120	0.01	0.90
	**TNW**	41.94 − 39.06	742.50	−0.553	0.06	0.58
	**NDW**	41.52 − 39.48	759.00	−0.395	0.04	0.69
	**MATTR**	34.97 − 41.11	818.00	1.222	0.14	0.22
	**MLU‐W**	45.78 − 35.22	589.00	−2.031	**0.23**	0.042*
	**MLU‐M**	44.75 − 36.25	630.00	−1.636	0.18	0.10
**Age 7**	**NTU**	−9.00	−0.660	18	0.31	0.518
	**TNW**	−45.00	−0.800	18	0.38	0.434
	**NDW**	−16.80	−0.916	18	0.43	0.372
	**MATTR**	−0.005	−0.173	15	0.09	0.865
	**MLU‐W**	0.134	0.381	18	0.18	0.707
	**MLU‐M**	12.40 − 8.60	31.00	−1.436	0.32	0.165
**Age 8**	**NTU**	11 − 10	45.00	−0.378	0.08	0.739
	**TNW**	11 − 10	45.00	−0.378	0.08	0.739
	**NDW**	16.300	0.586	18	0.28	0.565
	**MATTR**	0.0074	0.365	17	0.18	0.719
	**MLU‐W**	0.2160	1.065	18	0.50	0.301
	**MLU‐M**	0.3580	0.755	18	0.36	0.460
**Age 9**	**NTU**	12.30 − 8.70	32.00	−1.361	0.30	0.190
	**TNW**	13.35 − 7.65	21.50	−2.155	**0.48**	0.029*
	**NDW**	12.75 − 8.25	27.50	−1.703	0.38	0.089
	MATTR	8.20 − 12.00	63.00	1.476	0.33	0.156
	**MLU‐W**	0.552	2.198	18	**1.04**	0.041*
	**MLU‐M**	0.678	0.422	18	0.20	0.678
**Age 10**	**NTU**	9.80 − 11.20	57.00	0.530	0.12	0.631
	**TNW**	10.10 − 10.90	54.00	0.302	0.07	0.796
	**NDW**	−6.800	−0.189	18	0.09	0.852
	**MATTR**	−0.014	−0.713	18	0.34	0.485
	**MLU‐W**	0.045	0.130	18	0.06	0.898
	**MLU‐M**	0.068	0.110	18	0.05	0.914

*p* < .05; Mean difference, *t*, *df*, and effect size (*d*) are reported for independent samples *t*‐tests; Mean ranks, *U*, *z*, and effect size (*r*) are reported for Mann–Whitney *U* tests.

Among children aged 7 to 10, mean values of all microstructure measures except MATTR and median values of all measures except MATTR and NTU were higher for females (Table 4). In comparisons using the Mann–Whitney *U* test, a significant difference was observed only in the MLU‐W measure (Table 5); the median value for females was significantly higher (*p* = 0.042, *r* = 0.23), representing a small‐to‐moderate effect size.

In the age‐7 group, males showed higher mean and median scores in verbal productivity and semantic diversity measures, while females scored higher in syntactic complexity measures (Table 4). Group comparisons were conducted using the Mann–Whitney *U* test for MLU‐M and the independent samples *t*‐test for all other measures. No gender differences were observed across measures (*p* > 0.05) (Table 5).

According to Table 4, in the age‐8 group, both the median and mean values of MLU‐W and MLU‐M were higher for females. For NTU, TNW and NDW, mean values were higher for females, whereas median values were higher for males. In group comparisons, the Mann–Whitney *U* test was used for NTU and TNW, and the independent samples t‐test was used for the remaining measures. No statistically significant differences were found in any of the measures (*p* > 0.05) (Table 5).

In the age‐9 group, females exhibited higher mean and median scores across all microstructure measures except for MATTR (Table 4). Independent samples *t*‐tests were performed for MLU‐W and MLU‐M, while the Mann–Whitney *U* test was used for the remaining measures. Table 5 showed that the mean MLU‐W score for females was significantly higher than for males (*p* = 0.041, *d* = 1.04), representing a large effect size. Additionally, the median TNW score was significantly higher for females (*p* = 0.029, *r* = 0.48), representing a moderate‐to‐large effect size.

In the age‐10 group, mean and median values of semantic diversity measures were higher for males, while syntactic complexity measures were higher for females. In verbal productivity, the mean values were higher for females, whereas the median scores were higher for males (Table 4). Group comparisons were conducted using the Mann–Whitney *U* test for NTU and TNW and independent samples *t*‐tests for other measures. No significant gender differences were observed in microstructure measures (*p* > 0.05).

### 3.3 Frequency of Eliciting Personal Narratives Through Global TALES‐TR Prompts

To assess the effectiveness of the prompts in the protocol in eliciting personal narratives and to identify any possible difficulties children experienced, the frequency of narrative elicitation without follow‐up prompts and the frequency of prompts that did not elicit a narrative were calculated for each prompt.

#### 3.3.1 Children's Frequency of Follow‐Up Prompt Use in Global TALES‐TR Prompts

**FIGURE 2:**
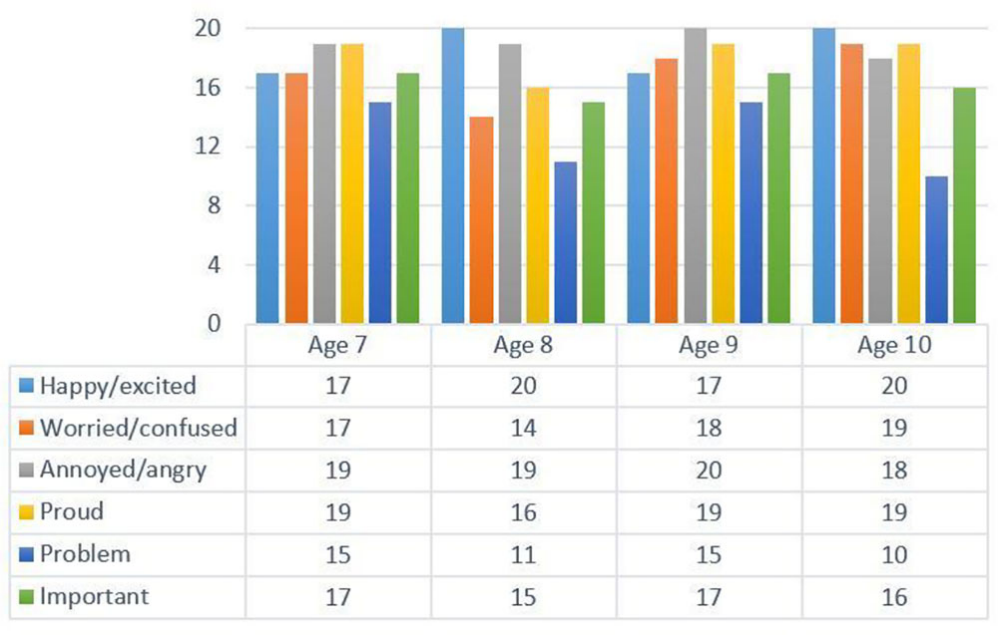
Frequency of narratives elicited without follow‐up prompts.

The frequency of children eliciting narratives without using follow‐up prompts for each prompt is presented in Figure 2.

As shown in Figure 2, the age‐7 group required the fewest follow‐up prompts for the ‘Annoyed/angry’ and ‘Proud’ narratives, while the most follow‐up prompts were needed for the ‘Problem’ narrative. An equal number of follow‐up prompts were used for the other narratives.

All participants in the age‐8 group elicited the ‘Happy/excited’ narrative without follow‐up prompts, while 45% of the children used follow‐up prompts during the ‘Problem’ narrative. Overall, the age‐8 group used the most follow‐up prompts across the entire protocol (Figure 2). Notably, four children in this group used follow‐up prompts in 3 out of 6 prompts, and one child used them in 4 out of 6 prompts.

In the age‐9 group, no follow‐up prompts were used in the ‘Annoyed/angry’ narrative, while the greatest need for follow‐up prompts was observed in the ‘Problem’ narrative. Across the protocol, the age‐9 group used the fewest follow‐up prompts (Figure 2). Additionally, one child required follow‐up prompts in 3 out of 6 prompts.

Although children in the age‐10 group did not use any follow‐up prompts in the ‘Happy/excited’ narrative, they were the group that used the most follow‐up prompts in the ‘Problem’ narrative among all age groups. In this prompt, 50% of the children used follow‐up prompts (Figure 2). Additionally, one child used follow‐up prompts for 3 out of 6 prompts, and another child used them for 4 out of 6 prompts.

**FIGURE 3:**
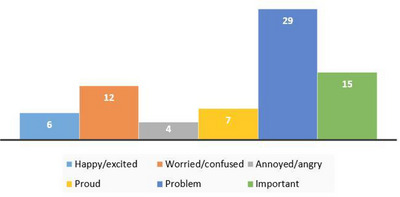
Frequency of follow‐up prompt use in children aged 7–10.

Frequencies of follow‐up prompt needs for six different prompts among all children (*n* = 80) are presented in Figure 3. Notably, the ‘Problem’ narrative required the highest number of follow‐up prompts, while the ‘Annoyed/angry’ narrative required the fewest.

#### 3.3.2 Frequency of Prompts That Did Not Elicit a Personal Narrative

**FIGURE 4:**
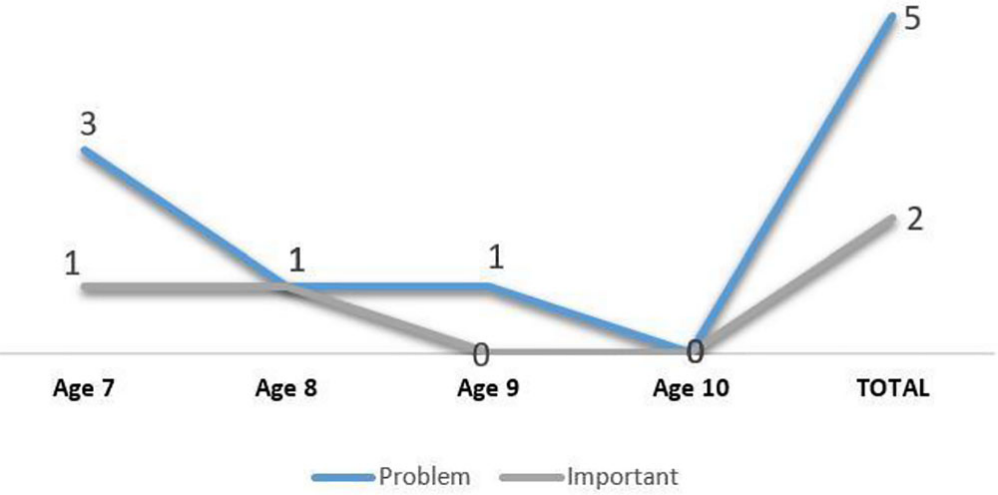
Frequency of unanswered prompts across age groups.

Despite using follow‐up prompts, five children did not respond in the ‘Problem’ narrative, including three children in the age‐7 group and one each in the age‐8 and age‐9 groups. Additionally, two children in the ‘Important’ narrative, one from the age‐7 and one from the age‐8 group, did not respond. Children in the age‐10 group responded to all prompts (see Figure 4).

### 3.4 Topics and Examples of Content Most Frequently Mentioned by Children in Narratives

Children's narrative topics and examples of content were examined for each prompt. In identifying the main topics, reference was made to studies that investigated narrative content using the Global TALES protocol (Lyons et al. 2023; Srivastava et al. 2023; Westby et al. 2023; Westerveld et al. 2022). For each prompt, the three narrative topics most frequently selected by children, along with representative content examples, are presented in Table 6.

**TABLE 6:** The three most frequent narrative topics per protocol prompt, with examples of their content.

	Topics	Examples	*f* ^*^
**Happy/excited**	Family event	Family trip, holiday, celebrating their own birthday	39
	Peer relationship	Time with friend, building new relationship	26
	New experience or item	Receiving a gift, enrolling in a swimming course	23
**Worried/confused**	School task	Not understanding, forgetting book, not getting homework done	44
	Not knowing how to do, what to do (not academic)	Being indecisive, struggling to make a choice, being unsure which way to go	15
	Safety concerns / fearful situation	Earthquake, being chased by a dog, being home alone, getting lost	14
**Annoyed/angry**	Sibling/peer relationship	Being blamed or excluded, having a cheating, lying, or rule‐breaking friend in the game, being bullied	63
	Personal frustration	Regret about not doing better, damaged item or toy, unable to find or do something	21
	Parental/adult issues	Permission refusal or plans cancelled, arguing with a parent, teacher not allowing a say	8
**Proud**	Personal achievement	Academic, sporting, or musical achievement, graduating	61
	Personal growth or contribution	Brave, honesty, helping someone, giving food to an animal	23
	Achievement involving others	Class success	2
**Problem**	Peer/family relationships	Resolving conflicts, differing opinions, standing up to bullies, friend shifting blame onto her/him	30
	Problem at school	Not understanding the lesson or solving questions, having trouble with project design	25
	Finding or fixing	Searching for a lost item, a clock breaking down, fixing a broken toy	10
**Important**	Personal achievement	School exams and homework, team or individual sports activities	29
	Safety/wellness/death	Injury, calling ambulance, falling down the stairs, earthquake	20
	Mishaps/personal loss/something being damaged	A cherished item being torn or broken	13

* *f* = frequency; Since some children elicited narratives on more than one topic, the total frequency may exceed the total number of participants.

## 4 Discussion

The Global TALES protocol was employed in this study to examine the personal narrative skills of TD children aged 7 to 10, a developmental stage during which children begin to categorise stories as ‘exciting’ and ‘funny’ (Larson and McKinley 1987; Paul 2002). Children's performance was examined based on verbal productivity, semantic diversity and syntactic complexity across the protocol prompts, as well as the narrative topics elicited. The results were discussed under subsections aligned with the research questions.

### 4.1 Microstructure Results from the Global TALES‐TR Narratives

NTU, a measure of verbal productivity, reflects children's willingness to produce personal narratives and their memory skills (Einarsdóttir and Þráinsdóttir 2023). A sample of approximately 50 utterances is generally accepted as sufficient for language sample analysis (Heilmann et al. 2010c; Miller 1981; Paul and Norbury 2012; Tilstra and McMaster 2007). In this study, 54% of the children produced at least 50 utterances across the protocol as a whole, and mean NTU values were close to or above this reference level. Individual variability was observed, consistent with previous Global TALES studies reporting that some children produce fewer utterances than the recommended threshold (Westerveld et al. 2022).

TNW reflects verbal productivity and fluency within a language sample (Acarlar 2024). Although TNW calculated from short samples is considered reliable (Tilstra and McMaster 2007), it does not capture semantic diversity; therefore, measures based on different word roots are also recommended (Miller 1981; Pezold et al. 2020). In this study, TNW scores ranged from 55 to 1366, while NDW scores ranged from 43 to 340. Comparable measures have been reported in previous Global TALES studies, with the current findings showing closer alignment with the Cypriot Greek sample (Einarsdóttir and Þráinsdóttir 2023; Theodorou et al. 2023). Whether this similarity reflects cultural factors warrants further investigation.

MATTR is another measure of semantic diversity that is independent of narrative length and considered more reliable than the type‐token ratio (Covington and McFall 2010). As a minimum of 100 words was required for calculation, MATTR could not be computed for five participants. Among the remaining children (*n* = 75), values ranged from 0.44 to 0.70, with only one child in the youngest age group falling below the reference value. Except for this child, all children with calculable MATTR values demonstrated sufficient semantic diversity. For this child, one narrative was deemed inappropriate, and another was left unanswered, which may have contributed to the lower MATTR due to limited NDW in the narratives included in the analysis. Notably, this parameter has not yet been investigated in other research using the same protocol.

MLU is another measure used in personal narratives, which can be calculated in words or morphemes, with a strong correlation between the two (Parker and Brorson 2005). Although previous Global TALES studies have primarily focused on MLU‐W (Einarsdóttir and Þraínsdóttir 2023; Lyons et al. 2023), both measures were included in this study due to the morphological significance of suffixes in Turkish (Ketrez and Aksu Koç 2003). In the present study, MLU‐W across age groups ranged from 3.45 to 6.53, with mean values between 4.67 and 4.97. Compared to findings from Ireland and Iceland, these values were lower but showed closer alignment with the Cypriot sample (Einarsdóttir and Þraínsdóttir 2023; Lyons et al. 2023). This likely reflects inherent morphological differences among languages, such as agglutinative versus inflectional types.

Microstructural measures showed individual variability across age groups. In the current study, variation in interview durations (5–22 min) may have contributed to this pattern. Similar within‐group variability has been reported in previous Global TALES studies (Einarsdóttir and Þráinsdóttir 2023; Lyons et al. 2023; Westerveld et al. 2022), suggesting that individual differences in microstructural outcomes are a consistent feature across countries, independent of language. This finding aligns with the pilot phase of the current study (Maviş and Yaşar‐Gündüz 2023). Individual variability, including children's reluctance to share personal experiences with an unfamiliar interlocutor, may also have contributed to variation in microstructure measures, as previously noted by Kuvač Kraljević et al. (2023).

#### 4.1.1 Age‐Related Differences in Microstructure Measures

Previous research indicates that TNW, NDW and MLU are sensitive to developmental change and generally increase with age (Ege et al. 1998; Klee 1992; Miller 1991). However, age‐related findings in personal narratives remain inconsistent, with some studies reporting no significant effects and others identifying differences across age groups (Hamdani et al. 2019; Westerveld and Vidler 2016; Westerveld et al. 2004; Westerveld et al. 2008). These inconsistencies may be attributed to differences in age ranges, analytic procedures and assessment tools. Using the Global TALES protocol, Kuvač Kraljević et al. (2023) reported a significant age effect for MLU‐W only between the youngest and oldest groups, whereas other microstructure measures showed a gradual age‐related increase. This pattern aligns with the gradual increases in NTU, TNW, NDW and MLU‐W observed in the current study. In contrast to the findings from the Turkish pilot study (Maviş and Yaşar‐Gündüz 2023), the present study identified some significant differences between the age‐7 and age‐10 groups, likely reflecting the larger sample size.

A significant difference in MATTR was observed between the age‐8 and age‐9 groups, which may be related to outlier NDW in the age‐9 group, as MATTR is derived from the NDW/TNW ratio. Using 0.50 as a reference (Miller 1991), mean MATTR scores for all age groups exceeded this threshold, with only one child falling below it.

MLU is sensitive to developmental changes (Ege et al. 1998; Parker and Brorson 2005); however, after reaching approximately 4–5, it becomes limited as a developmental indicator (Aksu‐Koç 1988). In the current study, no significant age‐related differences in MLU were found, consistent with previous research (Aksu‐Koç 1988; Maviş and Yaşar‐Gündüz 2023). Nevertheless, some children exhibited relatively high MLU values, supporting evidence that storytelling contexts may elicit increased syntactic complexity (Leadholm and Miller 1994; Wagner et al. 2000). Additionally, the gradual increase in MLU‐W and MLU‐M across most age groups observed in this study is consistent with reports of continued, incremental growth in MLU into adolescence and early adulthood (Nippold et al. 2005), as well as Turkish SALT reference data showing proportional increases with age (Acarlar 2024).

#### 4.1.2 Gender‐Related Differences in Microstructure Measures

Findings on gender differences in personal narratives vary. Previous studies have reported no significant gender differences in microstructure measures such as TNW, NDW and MLU (Hamdani et al. 2019; Vogindroukas et al. 2020). Consistent with these findings, gender‐related differences in the present study were limited in scope and effect, with significant differences favouring girls observed only in the age‐9 group (TNW and MLU‐W) and age 7–10 group (MLU‐W). These effects were age‐specific and did not appear to reflect a general gender pattern.

The findings are broadly consistent with evidence suggesting that girls may produce longer and more detailed personal narratives in middle childhood (Buckner and Fivush 1998); however, the limited number of significant effects in the present study warrant cautious interpretation. The TNW difference in the age‐9 group may result from females with outlier values. TNW is known to be strongly linked to MLU through its relation to skills such as utterance construction and length (Acarlar 2005). Thus, the observed group difference in MLU‐W is likely related to this TNW variation.

Overall, evidence on gender‐related differences in personal narrative performance remains limited. The present findings should therefore be interpreted as preliminary and context‐dependent rather than robust gender effects. Differences in age ranges, languages and cultural backgrounds across studies complicate direct comparisons, highlighting the need for further research.

### 4.2 Use of Follow‐Up Prompts in the Global TALES‐TR Protocol

Across all age groups, the ‘Problem’ narrative required the highest number of follow‐up prompts, indicating that it was the most challenging prompt. Notably, the highest rate of follow‐up prompt use was observed in the age‐10 group. Considering all participants, this prompt required the highest proportion of follow‐up prompts (36.25%). During data collection, some children responded with remarks like ‘Oh, this is hard’ or ‘Wait, I need to think about this’ when asked about this prompt. Similar patterns have been reported in other Global TALES studies, where the ‘Problem’ narrative elicited high rates of follow‐up prompts in samples from Brazil, Cyprus, New Zealand and India (Srivastava et al. 2023; Westerveld et al. 2022). This suggests that children generally tend to struggle with similar prompts regardless of language or country. However, further research is needed before making cultural or societal conclusions.

Across age groups, the fewest follow‐up prompts were required for the ‘Happy/excited’ and ‘Annoyed/angry’ narratives. Overall, these findings suggest that these prompts were easier for children to address compared to other narrative prompts. Consistent with the present findings, the ‘Annoyed/angry’ and ‘Happy/excited’ prompts were associated with the lowest rates of follow‐up prompt use across several languages and countries (Einarsdóttir and Þráinsdóttir 2023; Westerveld et al. 2022).

In the present study, overall follow‐up prompt use among children aged 7–10 ranged from 5% to 36.25%, aligning with rates reported in most Global TALES studies. Comparable ranges have been observed in samples such as New Zealand (5%–41.2%), whereas substantially higher rates in Cyprus (63.2%–94.4%) and the absence of follow‐up prompt use in Russia represent notable contrasts (Westerveld et al. 2022). These differences highlight the need to further investigate potential cultural or individual influences on follow‐up prompt use.

### 4.3 Prompts That Did Not Elicit Personal Narratives

The 473 narratives elicited using the adapted protocol indicate a high overall response rate (98.54%), regardless of follow‐up prompt use. Narratives that remained unanswered even after follow‐up prompts may provide insight into which prompts children found most challenging and inform use of the protocol.

Unanswered prompts were rare in the present study (1.45%) and were limited to the ‘Problem’ and ‘Important’ prompts, with most non‐responses occurring for the ‘Problem’ prompt. Missing responses were primarily observed in younger children, particularly those aged 7, whereas all prompts were successfully elicited from the age‐10 group. Similar patterns have been reported in other Global TALES studies, including lower response rates for the ‘Problem’ prompt in the Irish sample (Lyons et al. 2023) and higher non‐response rates for the ‘Important’ prompt in the feasibility study (Westerveld et al. 2022).

### 4.4 Narrative Topics Most Frequently Mentioned by Children

Children's personal narrative topics provide insight into what children consider important and reflect their perspectives, encouraging clinicians to move beyond purely language analysis (Westby et al. 2023). Previous research has shown that children's narratives often reflect their sociocultural norms, which may also influence microstructure measures (Bliss and McCabe 2008). Studies employing the Global TALES protocol have examined narrative topics to better understand these patterns.

Similarities in the most frequently mentioned topics were observed across studies using the Global TALES. In the present study, the top three topics for the ‘Annoyed/angry’ prompt were also reported in the Irish sample and the feasibility study (Lyons et al. 2023; Westerveld et al. 2022). Comparable overlap with the feasibility study was also found for the ‘Proud’ and ‘Problem’ prompts. Previous studies have similarly reported common primary topics across prompts (Srivastava et al. 2023; Westby et al. 2023). These findings suggest that, despite cultural differences, children tend to include comparable topics in their narratives. Variability in narrative content and topic frequency may reflect the flexibility afforded by the protocol (Westerveld et al. 2022).

The timing of protocol administration may influence the topics elicited. Data collection in the present study began in March 2023, coinciding with a major earthquake in Türkiye, and some children referred to this event in their ‘Worried/Confused’ and ‘Important’ narratives. Similarly, data collected during the COVID‐19 pandemic included pandemic‐related experiences in children's narratives (Lyons et al. 2023). Conversely, the Icelandic sample did not reference the pandemic despite data collection occurring during the same period (Einarsdóttir and Þráinsdóttir 2023).

Follow‐up prompts may influence narrative content. For instance, some children expanded their stories after hearing follow‐up prompts by saying, ‘Yes, me too,’ or ‘I can also talk about the exam.’

Another important consideration is that data were collected online. Both face‐to‐face and online administrations of the Global TALES have been reported in the literature and are considered appropriate. However, observational findings suggest that narrative content may differ by administration mode. For example, Ferman and Kawar (2023) noted that children in face‐to‐face settings tended to mention personal relationships and achievements involving more emotional responses, whereas in online settings, they referred to experiences with fewer emotions. Although these observations were not statistically tested, the online format may have influenced the narrative content.

## 5 Conclusion

This study examined the personal narratives of TD children aged 7–10, focusing on microstructural measures and narrative topics. Overall, limited age‐related differences were observed primarily in measures of verbal productivity and semantic diversity, with older children demonstrating higher performance. Gender‐related differences were limited, with higher MLU‐W and TNW observed for girls in specific age groups.

Follow‐up prompt use varied by prompt, with the highest frequency observed for the ‘Problem’ narrative and the lowest for the ‘Happy/excited’ and ‘Annoyed/angry’ narratives. Notably, even with follow‐up prompting, non‐responses occurred primarily for the ‘Problem’ prompt. Overall, these findings indicate that the ‘Problem’ prompt was the most challenging for children. Narrative topics largely aligned with previous studies, with children most frequently referring to family, friends, school and achievement.

This study extends the application of a cross‐cultural personal narrative assessment tool to Turkish. The findings indicate that the adapted protocol is feasible within the Turkish linguistic and cultural context. Importantly, the findings presented in this study should be interpreted as descriptive data and not as normative benchmarks. They are intended to inform future research and clinical interpretation and should not be used as diagnostic cut‐off values.

This study was conducted online with children aged 7–10, and the findings are limited to this sample. Age and gender were the primary variables examined, while other factors such as parental education and household income could not be analysed due to limited group distribution. Future research may compare online and face‐to‐face administrations, examine oral and written narratives together, and explore associations between personal narratives and factors such as temperament, reading experiences and family background. Including additional components, such as macrostructure measures, may further enrich narrative assessment. In addition, further studies may extend this work to diverse age and clinical groups using cross‐sectional or longitudinal designs.

## Funding

This research was supported by the Anadolu University Research Fund (BAP Number: 2212S201).

## Ethics Statement

Ethical approval for participating in the project team and using the Global TALES protocol in research has been granted by the Griffith University Human Research Ethics Committee (GUHREC, 2020/942) and the Anadolu University Scientific Research and Publication Ethics Committee of the Faculty of Health Sciences (Protocol No: 358190).

## Consent

Informed consent was obtained from all the participants.

## Conflicts of Interest

The authors have declared that no competing financial or nonfinancial interests existed at the time of publication.

## Supporting information




**Supporting file**: jlcd70219‐supp‐0001‐SuppMat.docx.

## Data Availability

The data sets used in this study are available from the corresponding author [EYG] upon reasonable request.
